# Hyperkalemia Brugada Sign: When Catheterization Lab Is Not the Answer

**DOI:** 10.5811/cpcem.2018.1.36979

**Published:** 2018-03-14

**Authors:** Rene M. Kukkamalla, Eric Katz

**Affiliations:** Maricopa Medical Center, Department of Emergency Medicine, Phoenix, Arizona

## CASE PRESENTATION

**Image 1:**
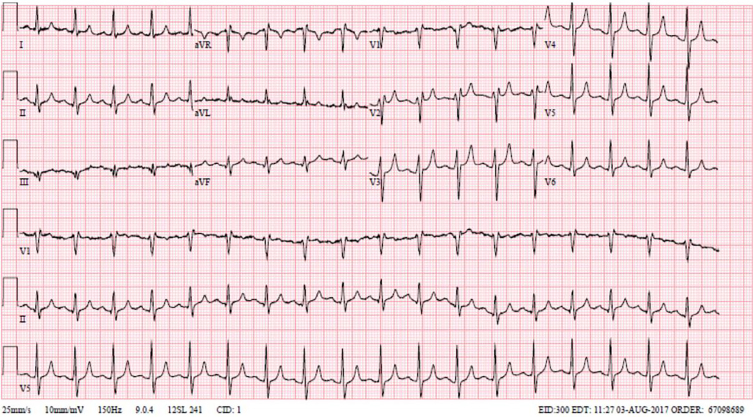
Initial electrocardiogram demonstrating peaked T-waves.

The patient was a 61-year-old male who presented to the emergency department (ED) with two days of emesis without abdominal pain. The patient’s initial electrocardiogram (EKG) showed normal sinus rhythm with peaked T-waves (Image 1).

**Image 2:**
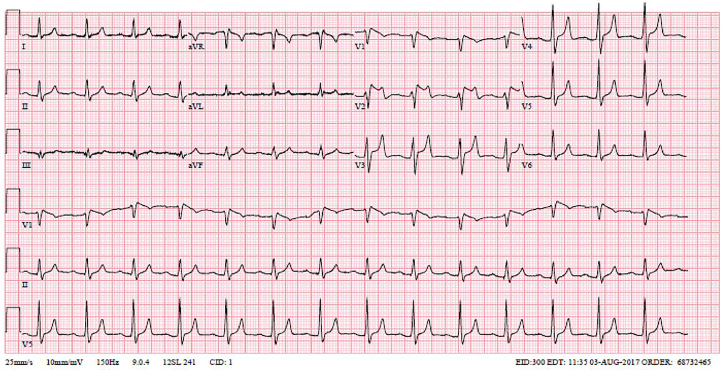
Repeat electrocardiogram demonstrating Brugada pattern.

Laboratory data were significant for hyperkalemia of 8.8 and new-onset renal failure with a creatinine of 7.2. A repeat EKG was obtained and showed an incomplete right bundle branch with Brugada pattern of ST-segment elevation in leads V1 and V2 (Image 2).

The automated EKG reading was an acute ST-segment elevation myocardial infarction; however, the lack of reciprocal changes made the ED team discount that. Cardiology was contacted for consultation and concurred, advising that the EKG change was likely secondary to hyperkalemia.

## DIAGNOSIS

This case represents hyperkalemia-induced Brugada pattern. Brugada syndrome is a congenital anomaly characterized by abnormities of the sodium ion channel. It often manifests as a widened QRS-segment on EKG with ST-segment elevation in the right precordial leads. A similar “Brugada pattern” (or “sign”) has been seen in the absence of this congenital sodium channel anomaly.

Hyperkalemia has multiple manifestations on EKG including peaked T-waves, QRS-segment prolongation, and sine wave pattern. It is important for emergency physicians to be familiar with less common manifestations as well, one of which includes the hyperkalemia-induced Brugada pattern. This change is reversible and typically resolves with resolution of hyperkalemia, which helps distinguish it from Brugada syndrome. Despite the ST-segment elevations anteriorly, patients with hyperkalemic Brugada sign would not benefit from the catheterization lab as the abnormality is from excess potassium, rather than a structural lesion.

**CPC-EM Capsule**

What do we already know about this clinical entity?

Brugada sign is usually associated with ischemia and sodium channel blockade. That it can be induced by hyperkalemia is less well known.

What is the major impact of the image(s)?

Recognition of hyperkalemic-induced Brugada sign is rare, and can save patients from unnecessary cardiac catheterization. In patients with hyperkalemia, the classic EKG changes are not the only ones to watch for.

How might this improve emergency medicine practice?

Early recognition helps separate two identical EKG presentations with different patient care needs.

Documented patient informed consent and/or Institutional Review Board approval has been obtained and filed for publication of this case report.
